# A Clinical Predictor Score for 30-Day Mortality among HIV-Infected Adults Hospitalized with Pneumonia in Uganda

**DOI:** 10.1371/journal.pone.0126591

**Published:** 2015-05-11

**Authors:** Catherine A. Koss, Leah G. Jarlsberg, Saskia den Boon, Adithya Cattamanchi, J. Lucian Davis, William Worodria, Irene Ayakaka, Ingvar Sanyu, Laurence Huang, International HIV-associated Opportunistic Pneumonias (IHOP) Study

**Affiliations:** 1 Department of Medicine, University of California, San Francisco, San Francisco, California, United States of America; 2 Division of Pulmonary and Critical Care Medicine, Department of Medicine, San Francisco General Hospital, University of California San Francisco, San Francisco, California, United States of America; 3 Makerere University–University of California San Francisco Research Collaboration, Kampala, Uganda; 4 Department of Medicine, Makerere University College of Health Sciences, Kampala, Uganda; 5 Department of Medicine, Mulago National Referral and Teaching Hospital, Kampala, Uganda; 6 HIV/AIDS Division, Department of Medicine, San Francisco General Hospital, University of California San Francisco, San Francisco, California, United States of America; Alberta Provincial Laboratory for Public Health/ University of Alberta, CANADA

## Abstract

**Background:**

Pneumonia is a major cause of mortality among HIV-infected patients. Pneumonia severity scores are promising tools to assist clinicians in predicting patients’ 30-day mortality, but existing scores were developed in populations infected with neither HIV nor tuberculosis (TB) and include laboratory data that may not be available in resource-limited settings. The objective of this study was to develop a score to predict mortality in HIV-infected adults with pneumonia in TB-endemic, resource-limited settings.

**Methods:**

We conducted a secondary analysis of data from a prospective study enrolling HIV-infected adults with cough ≥2 weeks and <6 months and clinically suspected pneumonia admitted to Mulago Hospital in Kampala, Uganda from September 2008 to March 2011. Patients provided two sputum specimens for mycobacteria, and those with Ziehl-Neelsen sputum smears that were negative for mycobacteria underwent bronchoscopy with inspection for Kaposi sarcoma and testing for mycobacteria and fungi, including *Pneumocystis jirovecii*. A multivariable best subsets regression model was developed, and one point was assigned to each variable in the model to develop a clinical predictor score for 30-day mortality.

**Results:**

Overall, 835 patients were studied (mean age 34 years, 53.4% female, 30-day mortality 18.2%). A four-point clinical predictor score was identified and included heart rate >120 beats/minute, respiratory rate >30 breaths/minute, oxygen saturation <90%, and CD4 cell count <50 cells/mm^3^. Patients’ 30-day mortality, stratified by score, was: score 0 or 1, 12.6%, score 2 or 3, 23.4%, score 4, 53.9%. For each 1 point change in clinical predictor score, the odds of 30-day mortality increased by 65% (OR 1.65, 95% CI 1.39-1.96, p <0.001).

**Conclusions:**

A simple, four-point scoring system can stratify patients by levels of risk for mortality. Rapid identification of higher risk patients combined with provision of timely and appropriate treatment may improve clinical outcomes. This predictor score should be validated in other resource-limited settings.

## Introduction

Pulmonary complications are a major cause of morbidity and mortality among HIV-infected patients worldwide. In spite of the increasing availability of effective combination antiretroviral therapy (ART), pneumonia and tuberculosis (TB) remain major causes of mortality in HIV-infected patients in resource-limited settings.[1, 2] Several studies have shown that mortality is higher in HIV-infected patients with pneumonia than in HIV-uninfected patients.[3–7] Moreover, in high-income countries such as Denmark, in which ART is now widely available, the risk of first-time hospitalization for pneumonia has been shown to be six-fold higher in HIV-infected persons compared to the general population, and hospitalization for pneumonia is associated with an increased mortality for the following year.[8, 9] Given the high mortality of pneumonia in HIV-infected patients, the development of a clinical predictor score for mortality in HIV-infected persons hospitalized for pneumonia could be an important clinical tool.

Previous studies have developed pneumonia severity scores, such as the Pneumonia Severity Index (PSI) and the CURB-65, to predict mortality in patients with pneumonia in the general population.[10, 11] However, these studies were conducted in high-income countries in patients that were infected with neither HIV nor TB. Furthermore, these studies included laboratory data, such as arterial blood gas, that may not be available in resource-limited settings where the majority of HIV-infected persons reside. Several studies have investigated the use of pneumonia severity scores in HIV-infected patients in high-income countries. One study evaluated the American Thoracic Society criteria for pneumonia in HIV-infected patients in Spain, and studies from the United States and Canada have evaluated the use of the PSI in patients with HIV.[12–14] The PSI has also been studied in low-income countries, although patients with HIV and TB were excluded.[15] A prior study conducted in the United States developed a staging system to predict mortality in HIV-infected patients with pneumonia but excluded patients with a history of TB.[16] A severity score has been developed for use in HIV-infected children with respiratory infections in resource-limited settings.[17] However, little has been done to develop prognostic tools in HIV-infected adult patients hospitalized with pneumonia in resource-limited, TB-endemic settings, particularly in sub-Saharan Africa. Thus, the aim of this study was to develop a clinical score using data that are often available at the time of initial evaluation to predict 30-day mortality in HIV-infected adults hospitalized with pneumonia in a TB-endemic and resource-limited setting.

## Methods

### Study Population

We conducted a secondary analysis of a prospective cohort study of opportunistic pneumonias enrolling patients who were admitted to Mulago Hospital, the national referral hospital located in Kampala, Uganda, from September 2008 to March 2011. The study’s primary focus was on HIV-associated TB and *Pneumocystis* pneumonia (PCP), and so we enrolled consecutive adults at least 18 years of age with suspected HIV, cough for at least 2 weeks but fewer than 6 months, and clinically suspected pneumonia. All subjects provided written informed consent. Patients with a reduced level of consciousness were not enrolled, as these patients could not be consented for the study. Patients who were already receiving treatment for TB or who tested negative for HIV infection were excluded from this analysis.

### Data Collection

Enrolled participants underwent a standardized evaluation of their respiratory symptoms. Demographic data were collected using a standardized patient questionnaire. Clinical data gathered included symptoms and signs of pneumonia and clinical characteristics available for most patients at the time of presentation. Patients without a known, confirmed HIV diagnosis were tested for HIV infection. HIV-infected patients had a chest radiograph and CD4 cell count measurement performed. Patients provided two sputum specimens for smear examination with Ziehl-Neelsen staining for acid-fast bacilli (AFB), per World Health Organization (WHO) guidelines recommending the collection of two, rather than, three sputa.[18] For additional diagnostic yield, patients with sputum smears that were negative for mycobacteria were referred for bronchoscopy with bronchoalveolar lavage (BAL). Mulago Hospital has personnel and equipment to perform bronchoscopy and this test is available to clinicians. Bronchoscopic inspection for Kaposi sarcoma (KS) was performed and BAL fluid was tested for mycobacteria, *Pneumocystis jirovecii* (modified Giemsa stain), and other fungi (potassium hydroxide smear, India ink stain, and culture on Sabouraud agar).

Diagnosis of TB was based on positive sputum or BAL culture on Lowenstein-Jensen media, which was performed throughout the study, or detection by mycobacterial growth indicator tube (MGIT), which was performed on specimens starting in May 2009, or Gene Xpert (Cepheid, Sunnyvale, CA), which was performed starting in August 2009. Diagnosis of fungal pneumonia was based on a positive BAL fungal culture. Diagnosis of PCP was based on microscopic visualization of the characteristic *Pneumocystis* cysts and trophic forms on Diff-Quik-stained BAL specimens. Diagnosis of pulmonary KS was based on visualization of the characteristic KS lesions during bronchoscopic inspection of the tracheobronchial tree. Cases were reviewed by study physicians at two months and after microbiologic results were known and final diagnoses were assigned according to standardized criteria. In addition to the above diagnostic classification, patients who improved after taking TB medications, in whom no alternate diagnosis was found, and whose AFB cultures were negative were presumed to have had culture-negative TB. Patients who had pulmonary infiltrates on chest radiography, improved after taking antibiotics and no other antimicrobials, and in whom no alternate diagnosis was found were presumed to have had bacterial pneumonia. Patients were evaluated either in-person or by telephone at two months to determine their vital status. Patients who were lost to follow-up are listed as having an unknown final diagnosis.

### Statistical Analysis and Derivation of the Clinical Predictor Score

All statistical analyses were performed using SAS 9.2 (SAS Institute, Cary, North Carolina, USA). Vital sign cutoffs entered into the model were selected based on clinically meaningful values. Univariate associations between demographic and clinical variables and 30-day mortality were tested using the χ^2^ test; risk ratios (RR) and 95% confidence intervals (CI) were calculated using the Mantel-Haenszel method. Statistically or clinically significant variables were retained in a stepwise sequence of models using a logistic procedure. Akaike information criterion (AIC) and Schwarz information criterion (SC) scores were used to determine the optimal number of variables to include in the most predictive model. A best subsets logistic regression was then performed to identify the model with the optimal number of variables based on AIC and SC scores that had the highest χ^2^ value.[19] We used the bootstrap method to simulate 200 populations with the number of subjects in our cohort. One point was assigned to each variable in the final model to develop a clinical predictor score for 30-day mortality. The score’s ability to predict mortality in this cohort was assessed using logistic regression with score as a continuous factor (values ranging from 0 to 4) and then as a categorical factor (values grouped into 3 categories: 0–1, 2–3, and 4) for simplification of risk strata into lower, intermediate, and higher risk. The calibration of the predictor score was evaluated by the Hosmer-Lemeshow goodness of fit test and discrimination was evaluated by determining the proportion of patients in each score category among those who died and among those who survived.[20]

### Ethics Approval

The Institutional Review Board at Mulago Hospital, the Makerere University School of Medicine Research Ethics Committee, the Uganda National Council for Science and Technology, and the Committee on Human Research at the University of California, San Francisco approved the study protocol.

## Results

### Patient Characteristics

**Fig 1:**
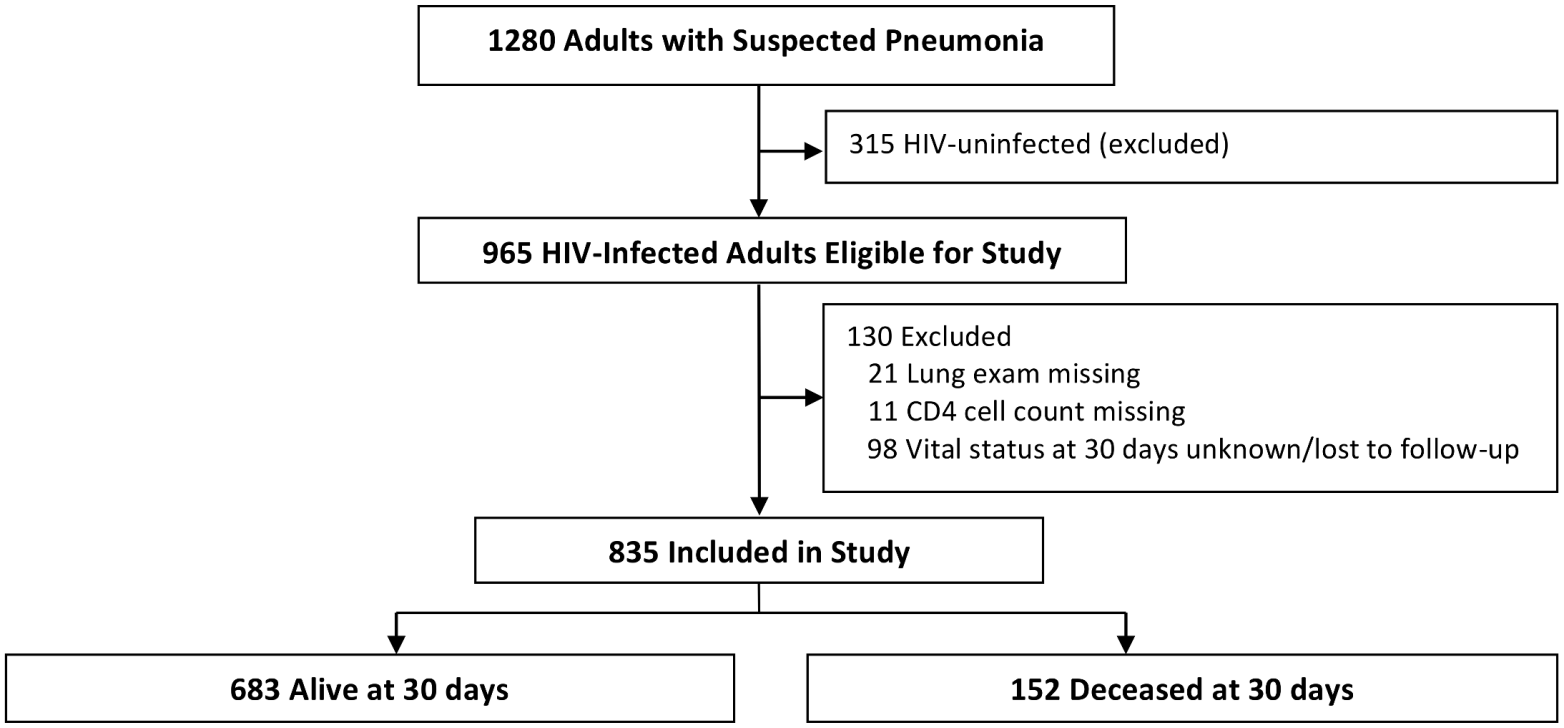
Screening and follow-up of study participants.

Of 1280 patients enrolled in the study, 965 were infected with HIV and therefore eligible for this study (Fig 1). We excluded HIV-infected patients who had a missing lung exam (21 patients) or a missing CD4 cell count (11 patients). We included HIV-infected patients who died within 30 days but excluded those who had missing data on vital status at 30 days or were lost to follow-up (98 patients). Baseline characteristics (e.g., age, proportion on ART at enrollment, proportion on PCP prophylaxis at enrollment) were not significantly different between the included and excluded patients with the exception of baseline CD4 cell count (lower in enrolled subjects) and sex (more men in enrolled subjects).

The included study population of 835 HIV-infected patients was predominantly female (53.4%) and below the age of 45 years (87.5%) (Table 1). HIV was newly diagnosed in 256 patients (30.7%). The majority of patients reported fever, chills, or night sweats (88.1%), subjective weight loss (95.5%), sputum production (91.7%), dyspnea (52.2%), and chest pain (64.6%). Only 13.7% of patients had been on ART for at least 30 days prior to enrollment and 44.2% had been taking PCP prophylaxis for at least 30 days prior to enrollment. Overall, 39% of patients had a temperature below 35.5°C or above 38°C, 27.8% had a heart rate above 120 beats/minute, 45.3% had a respiratory rate above 30 breaths/minute, and 16.4% had an oxygen saturation below 90% on room air at the time of study enrollment. Most patients (62.4%) had an abnormal lung examination. Nearly half (49.6%) of patients had a CD4 cell count below 50 cells/mm^3^ at enrollment. Over half (51.3%) of patients were diagnosed with pulmonary TB, whereas 16 (1.9%) had pulmonary Kaposi sarcoma, 11 (1.3%) had PCP, and 80 (9.6%) patients were presumed to have had bacterial pneumonia.

**Table 1:** Baseline characteristics and final diagnoses of 835 subjects.

Characteristic	n (%)
Female	446 (53.4)
Male	389 (46.6)
Age	
< 25 years	111 (13.3)
25–34 years	369 (44.2)
35–44 years	251 (30.1)
≥ 45 years	104 (12.5)
New HIV diagnosis	256 (30.7)
Fever, chills, or night sweats	736 (88.1)
Weight loss (subjective)	797 (95.5)
Cough*	835 (100.0)
Sputum production	766 (91.7)
Dyspnea	436 (52.2)
Wheezing	201 (24.1)
Chest pain	539 (64.6)
Smoked > 99 cigarettes in lifetime	191 (22.9)
Ever consumed alcohol	544 (65.2)
ART ≥ 30 days prior to enrollment	114 (13.7)
PCP prophylaxis ≥ 30 days prior to enrollment	369 (44.2)
Temperature <35.5°C or >38°C	326 (39.0)
Heart rate >120 beats/minute	232 (27.8)
Respiratory rate >30 breaths/minute	378 (45.3)
Oxygen Saturation <90%	137 (16.4)
Abnormal lung exam**	521 (62.4)
CD4 cell count <50 cells/mm^3^	414 (49.6)
**Diagnosis** ***	
Pulmonary tuberculosis	429 (51.3)
*Pneumocystis* pneumonia	11 (1.3)
Pulmonary Kaposi’s sarcoma	16 (1.9)
Presumed bacterial pneumonia	80 (9.6)
Other	27 (3.2)
Unknown	277 (33.2)

* Cough was a criterion for study inclusion.

** Rhonchi, crepitations, or bronchial breath sounds.

*** Four subjects had more than one diagnosis: two had pulmonary tuberculosis (TB) & *Pneumocystis* pneumonia, one had pulmonary TB & pulmonary Kaposi’s sarcoma (KS), one had pulmonary KS & *Pneumocystis* pneumonia, and one had pulmonary KS and presumed bacterial pneumonia.

ART, antiretroviral therapy; PCP, *Pneumocystis* pneumonia.

### Mortality

At 30 days, 152 (18.2%) patients had died (Table 2), the majority (57%) of whom died within the first 10 days of their admission. Significantly more patients died who presented with dyspnea (21.1% vs. 15.0%, p = 0.02), heart rate above 120 beats/minute (23.7% vs. 16.1%, p = 0.01), respiratory rate above 30 breaths/minute (23.3% vs. 14.0%, p = 0.0005), and oxygen saturation below 90% (31.4% vs. 15.6%, p <0.0001). Among patients with a CD4 cell count below 50 cells/mm^3^, 22.7% died compared to 13.8% of patients with a CD4 cell count of 50 cells/mm^3^ or above (p = 0.0008). More patients with a temperature below 35.5°C or above 38°C also died as compared to normothermic patients, although this difference was not statistically significant (20.9% vs. 16.5%, p = 0.11). There was a trend toward lower mortality among patients on ART for at least 30 days prior to enrollment and patients taking PCP prophylaxis for at least 30 days prior to enrollment but neither association was statistically significant.

**Table 2:** Univariate analysis of the association between selected clinical characteristics and 30-day mortality.

Clinical Characteristic	Died			
	n (%)	RR	95% CI	p-value
All subjects	152 (18.2)			
Female	77 (17.3)	0.89	0.67–1.19	0.45
Mean age (SE)	33.7 ±8.5	0.90	0.74–1.09	0.28
New diagnosis of HIV	43 (16.8)	0.89	0.65–1.23	0.48
Fevers, chills, night sweats	130 (17.7)	0.79	0.53–1.19	0.27
Weight loss (subjective)	148 (18.6)	1.76	0.69–4.51	0.21
Sputum production	140 (18.2)	1.05	0.62–1.80	0.86
Dyspnea	92 (21.1)	1.40	1.04–1.89	0.02
Wheezing	37 (18.4)	1.01	0.73–1.42	0.93
Chest pain	96 (17.8)	0.94	0.70–1.27	0.69
Smoked >99 cigarettes (lifetime)	37 (19.4)	1.08	0.78–1.51	0.63
Ever consumed alcohol	107 (19.7)	1.27	0.93–1.75	0.13
ART ≥ 30 days prior to enrollment	18 (15.8)	0.85	0.54–1.33	0.47
PCP prophylaxis ≥ 30 days prior to enrollment	65 (17.6)	0.94	0.71–1.26	0.70
Temperature <35.5°C or >38°C	68 (20.9)	1.26	0.95–1.69	0.11
Heart rate >120 beats/minute	55 (23.7)	1.47	1.10–1.98	0.01
Respiratory rate >30 breaths/minute	88 (23.3)	1.66	1.24–2.23	0.0005
Oxygen saturation <90%	43 (31.4)	2.01	1.49–2.72	<0.0001
Abnormal lung exam	101 (19.4)	1.19	0.88–1.62	0.25
CD4 cell count <50 cells/mm^3^	94 (22.7)	1.65	1.22–2.22	0.0008

RR, risk ratio; CI, confidence interval; SE, standard error; PCP, *Pneumocystis* pneumonia.

### Derivation of the Clinical Predictor Score

The primary outcome of this study was 30-day mortality. Variables included in the derivation of the clinical predictor score were: use of ART for at least 30 days prior to enrollment, use of PCP prophylaxis for at least 30 days prior to enrollment, temperature below 35.5°C or above 38°C, heart rate above 120 beats/minute, respiratory rate above 30 breaths/minute, oxygen saturation below 90%, and CD4 cell count below 50 cells/mm^3^. A separate model included the above variables and age and cigarette use, which had no effect on the variables included in the predictor score. Minimum AIC and SC scores indicated that a model with 3 to 5 variables would be optimal. Best subsets regression showed that the best model with 3 variables was: respiratory rate above 30 breaths/minute, oxygen saturation below 90%, and CD4 cell count below 50 cells/mm^3^ (χ^2^ = 35.1). The best model with 4 variables included these 3 variables with the addition of heart rate above 120 beats/minute and showed substantial improvement over the 3-variable model (χ^2^ = 38.1). The best models with 5 variables included all variables in the 4-variable model but no single fifth variable offered a substantial gain in predictive value over the 4-variable model (χ^2^ = 38.3 to 38.5 for all 5-variable models); thus, the 4-variable model was selected. The bootstrap method was then used to simulate 200 populations each comprised of 835 subjects. Based on the best subsets regression analysis, we identified a 4-variable clinical predictor score that included heart rate above 120 beats/minute, respiratory rate above 30 breaths/ minute, oxygen saturation below 90%, and CD4 cell count below 50 cells/mm^3^. One point was assigned for each clinical predictor present, with a range of scores from 0 to 4.

### Association between Score and Mortality

**Fig 2:**
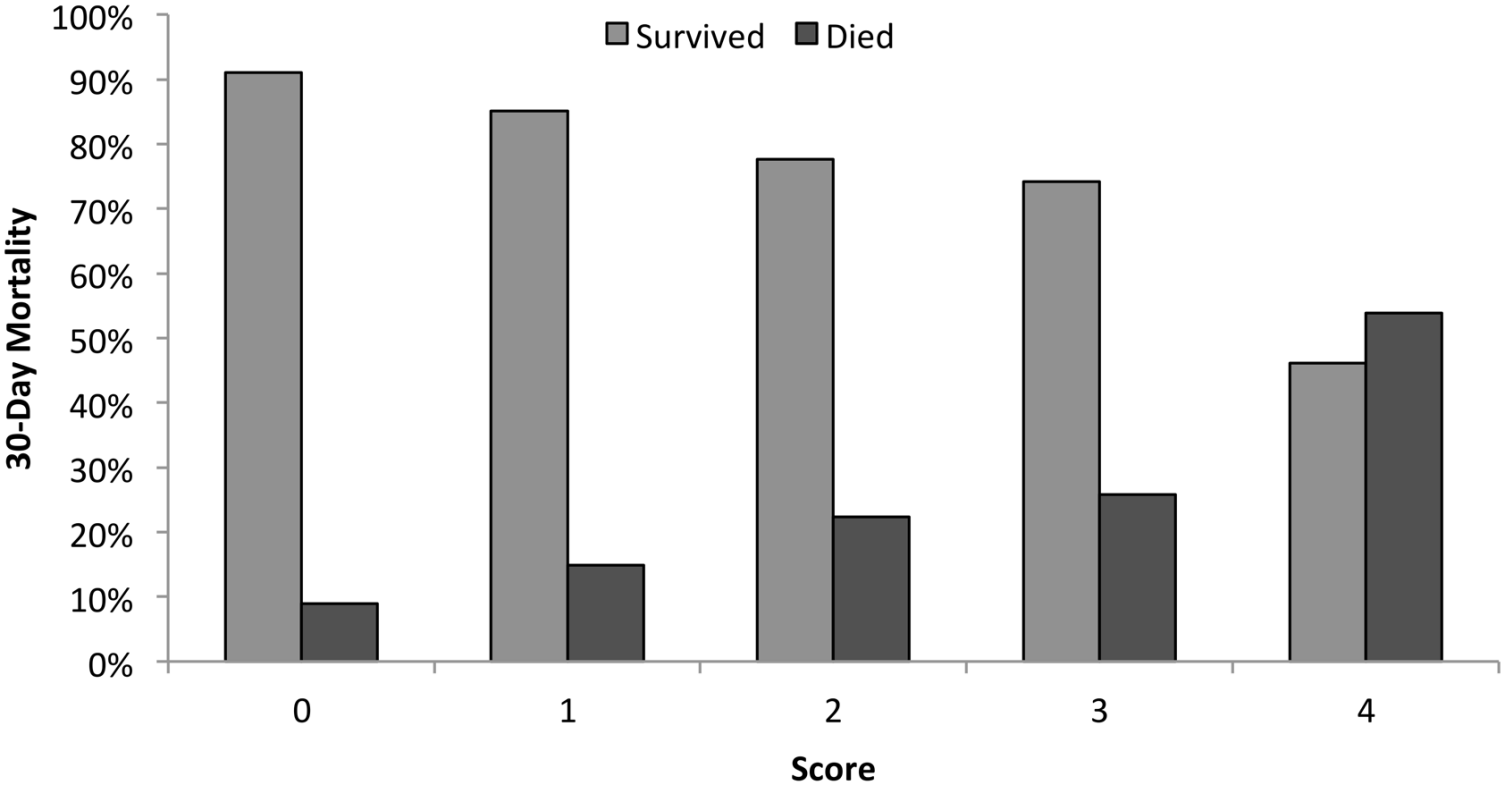
30-day vital status for patients with each clinical predictor score. p <0.0001 for the difference in mortality across clinical predictor score values.

**Fig 3:**
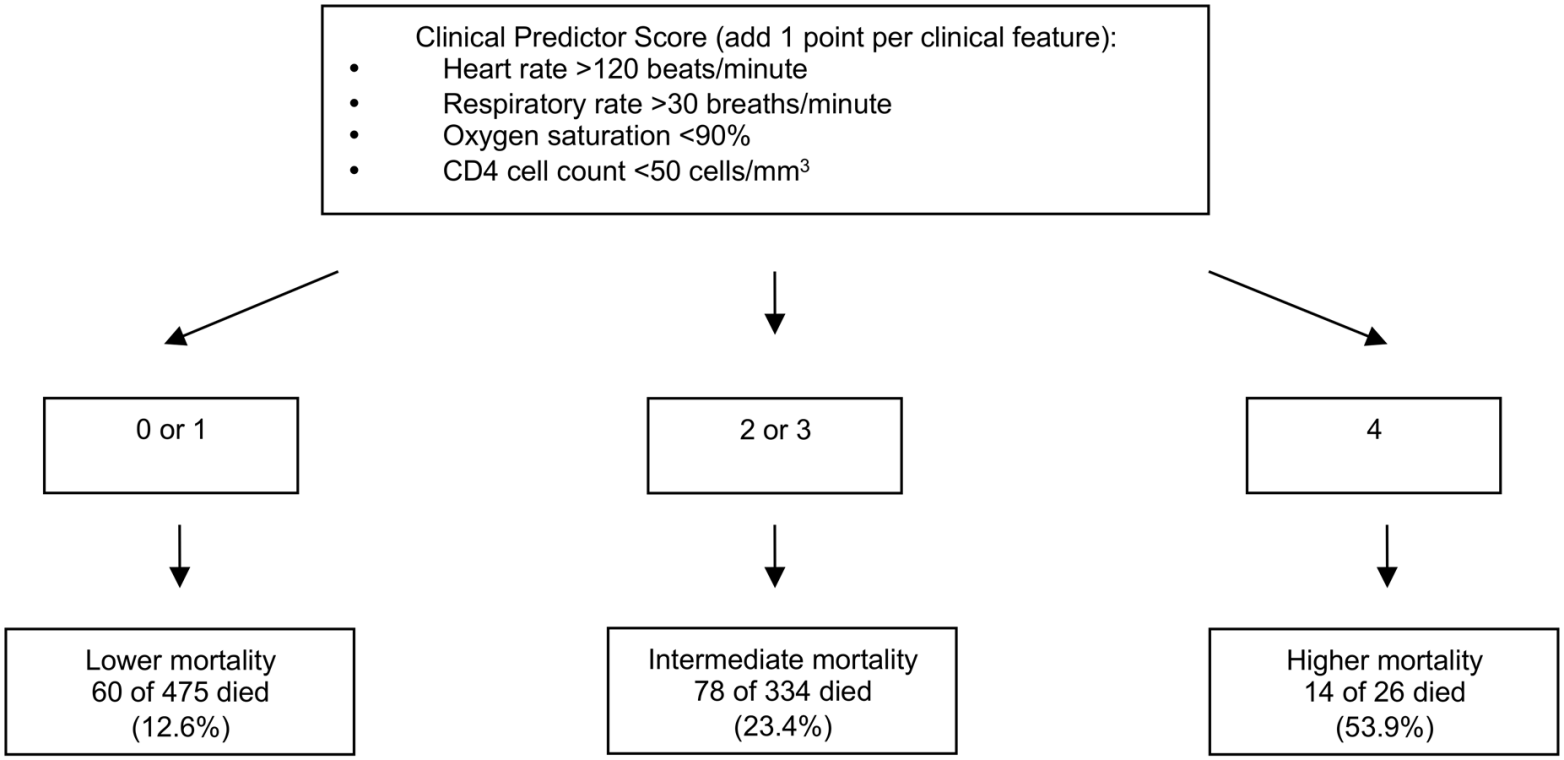
Stratification of patients’ 30-day risk of mortality based on clinical predictor score.

Patients with higher clinical predictor scores had higher mortality rates at 30 days (p <0.0001), (Fig 2). We stratified patients into groups of increasing risk for mortality based on score (Fig 3). Among patients with a clinical predictor score of 0 or 1, 12.6% had died at 30 days, as compared to 23.4% of patients with a score of 2 or 3, and 53.9% of patients with a score of 4 (p <0.001 for lower mortality vs. intermediate mortality vs. higher mortality groups). For each 1 point change in clinical predictor score, the odds of 30-day mortality increased by 65% (OR 1.65, 95% CI 1.39–1.96, p <0.001).

The calibration of the predictor score was evaluated by the Hosmer-Lemeshow test, χ^2^ = 1.12 (p = 0.98), indicating that this model fit the data well. The discrimination of the predictor score was evaluated by determining the proportion of patients in each score category among those who died and among those who survived. Among patients who died, the percentage of patients with each score was as follows: 0 (10.5%), 1 (28.9%), 2 (35.5%), 3 (15.8%), 4 (9.2%). Among patients who survived, the percentage with each score was: 0 (23.9%), 1 (36.9%), 2 (27.4%), 3 (10.1%), 4 (1.8%).

## Discussion

This study of HIV-infected patients admitted to an East African referral hospital with pneumonia and a high frequency of TB found that mortality was high overall and that a clinical predictor score using data that are often available at the time of initial evaluation can be used to stratify patients by levels of risk for mortality. We found an overall mortality rate of 18.2% at 30 days among patients in this study. This high mortality rate highlights the importance of rapidly identifying patients at an increased risk of death and targeting those individuals for timely interventions.

In our study, we identified four clinical predictors (heart rate above 120 beats/minute, respiratory rate above 30 breaths/minute, oxygen saturation below 90%, and CD4 cell count below 50 cells/mm^3^) that were combined into a predictor score that stratifies patients by levels of risk for mortality. The clinical predictor score developed in our study can be implemented with limited laboratory data, in contrast to prior scoring systems, which often require laboratory parameters that may not be readily available in resource-limited settings. For example, CURB-65 requires the measurement of uremia, although a simplified version, CRB-65, does not require the use of this laboratory data.[11] The PSI incorporates 20 parameters, including arterial blood gas, and a score to predict mortality due to HIV-associated PCP makes use of the alveolar-arterial (A-a) oxygen gradient.[10, 21] Rather than requiring the use of blood gases, our clinical predictor score incorporates the measurement of oxygen saturation with a pulse oximeter. Other studies have shown that low oxygen saturation is a risk factor for mortality; for example, oxygen saturation below 90% was significantly associated with in-hospital mortality among HIV-infected patients with pneumonia in the United States.[16] Oxygen supplies are often inconsistently available in resource-limited settings. Given the strong association between hypoxemia and mortality in this study, patients may benefit from routine evaluation of oxygen saturation at the time of triage and from the provision of supplemental oxygen to those with a low saturation. Our study also highlights the need for expanded oxygen delivery capability in similar settings. Moreover, this relatively low cost investment could reap significant benefits with regard to patient outcomes.

A CD4 cell count below 50 cells/mm^3^ was significantly associated with increased 30-day mortality and is also a component of this clinical predictor score. Low CD4 cell count has been shown to be associated with higher rates of mortality in prior studies of HIV-infected patients with pneumonia, including a study of patients with bacterial pneumonia conducted in Spain.[22] Point of care CD4 testing enables rapid testing of patients upon presentation with results available within 20 minutes, has been shown to be feasible in pilot testing, and is increasingly becoming available in resource-limited settings.[23] Given the strong association between low CD4 cell count and risk of mortality in this cohort, increased availability of point of care CD4 testing could help to rapidly identify patients at increased risk of mortality.

In our study, although nearly 70% of patients had been diagnosed with HIV prior to enrollment and nearly half had a CD4 cell count below 50 cells/mm^3^, only 18.7% of patients who were aware of their HIV status were on ART for at least 30 days prior to entry. At the time that this study began enrollment, national antiretroviral treatment guidelines for Uganda recommended ART initiation for all HIV-infected patients with a CD4 cell count below 200 cells/mm^3^; patients with a CD4 cell count between 200 and 350 cells/mm^3^ who were co-infected with TB, pregnant, or had severe bacterial infection (e.g. requiring hospitalization); and in patients with WHO Stage IV disease irrespective of CD4 cell count.[24] Since the completion of this study, in 2013, the WHO recommended ART initiation in all individuals with a CD4 cell count less than 500 cells/mm^3^, and in December 2013, the Ugandan antiretroviral treatment guidelines raised the CD4 threshold to less than 500 cells/mm^3^, in line with WHO guidelines.[25] [26]

The significance of CD4 cell count as a predictor of 30-day mortality in our study raises the question of whether the prompt initiation of ART, and subsequent improvement in CD4 cell count, could improve outcomes in this population. A previous study showed that starting ART within 14 days of diagnosis of an acute opportunistic infection (OI) other than TB resulted in less AIDS progression and death versus starting ART after completion of treatment for an OI.[27] The majority of subjects were enrolled in the United States; 63% of patients had PCP and 12% had bacterial infections including pneumonia. The CAMELIA, SAPIT, and STRIDE studies showed that treatment of HIV and pulmonary TB improves survival and reduces HIV-related illnesses.[28–30] The STRIDE trial showed a significant reduction in AIDS-defining illness or death among patients with a CD4 cell count below 50 cells/mm^3^ in patients who started ART within two weeks of initiation of treatment for tuberculosis.[28] TB was the most common diagnosis in our cohort, which is consistent with prior studies showing that TB is a leading cause of death among HIV-infected patients in sub-Saharan Africa.[31, 32] Our data reflect the persistent need for timely diagnosis of HIV infection and earlier initiation of ART, which may also reduce rates of TB and other HIV-related complications in this patient population.

Strengths of this study include the prospective nature, the standardized diagnostic evaluation and 30-day outcome assessment, and the large cohort size, which improved the study’s power. To our knowledge, this is the only clinical predictor score for pneumonia that has been developed in adult patients with HIV from a resource-limited setting with a high prevalence of TB. The relatively young age of patients in this study is representative of the HIV-infected population in Uganda, as well as Uganda’s demographics as a whole.[33] A limitation of this study is that we only enrolled patients admitted to a single referral hospital. Although this is a single site study, the size of the study population (835) is comparable to the initial studies of well-validated pneumonia severity scores. The CURB-65 investigators studied 1068 patients at three sites.[11] The Pneumonia Severity Index was initially derived from a study of 347 patients at three Pittsburgh hospitals, and later went on to be validated and refined using hospital databases containing thousands of patient records. [10, 34–36] Our standardized clinical and diagnostic evaluation (e.g., bronchoscopy) and follow-up in a resource-limited setting are strengths of the study, and we hope that our results are validated in additional settings much like the initial Pneumonia Severity Index study was subsequently validated. Furthermore, as we are currently enrolling participants in another cohort study in Uganda of HIV-infected adults with lung disease, we will be able to test and validate this score on those participants. Whereas our clinical predictor score would be expected to perform well in individuals with a subacute presentation of cough, future studies should be conducted to validate our findings in HIV-infected patients with respiratory complaints of shorter duration (i.e. less than 2 weeks) than those in our study. Predictive models generally perform better in the specific population in which they were developed; thus, this clinical predictor score should also be validated in similar populations and in other settings. Another potential limitation of the study is that approximately 10% of patients did not have complete information on vital status at 30 days despite attempts by study staff to contact these individuals.

Identifying patients at high risker for short-term mortality may allow for appropriate allocation of additional targeted interventions in resource-limited settings. These interventions may include the use of diagnostic testing (such as GeneXpert for rapid TB diagnosis) and therapeutics (including early goal directed therapy for sepsis, supplemental oxygen for hypoxemic patients, and ensuring early ART for all patients). Mortality was high overall in this cohort and further research is needed to optimize both diagnostic and therapeutic strategies in this population in order to improve outcomes and reduce mortality.

In conclusion, we found that a simple, four-point scoring system can stratify HIV-infected patients with pneumonia by increasing levels of risk for 30-day mortality. The rapid identification of patients at an increased risk for short-term mortality, combined with timely and appropriate diagnosis and treatment, may improve clinical outcomes. With further validation in other clinical settings, this clinical predictor score may help to improve the management of pneumonia in HIV-infected patients in resource-limited settings.
